# (*E*)-3-Bromo-*N*′-(4-methoxy­benzyl­idene)benzohydrazide methanol solvate

**DOI:** 10.1107/S1600536809030219

**Published:** 2009-08-08

**Authors:** Guo-Biao Cao

**Affiliations:** a Department of Chemistry, Ankang University, Ankang Shanxi 725000, People’s Republic of China

## Abstract

The title compound, C_15_H_13_BrN_2_O_2_·CH_3_OH, was synthesized by the reaction of 4-methoxy­benzaldehyde with an equimolar quantity of 3-bromo­benzohydrazide in methanol. The benzohydrazide mol­ecule displays an *E* configuration about the C=N bond. The dihedral angle between the two benzene rings is 4.0 (2)°. The benzohydrazide and methanol mol­ecules are linked into a chain propagating along the *b* axis by O—H⋯O, O—H⋯N, N—H⋯O and C—H⋯O hydrogen bonds.

## Related literature

For the crystal structures of hydrazone compounds, see: Mohd Lair *et al.* (2009 ▶); Fun *et al.* (2008 ▶); Li & Ban (2009 ▶); Zhu *et al.* (2009 ▶); Yang (2007 ▶); You *et al.* (2008 ▶). For hydrazone compounds reported previously by our group, see: Qu *et al.* (2008 ▶); Yang *et al.* (2008 ▶); Cao & Lu (2009*a* ▶,*b* ▶); Qu & Cao (2009 ▶); Cao & Wang (2009 ▶); Cao (2009 ▶). 
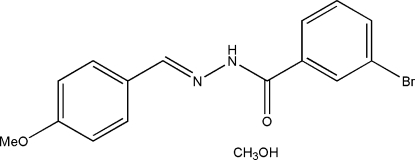


## Experimental

### 

#### Crystal data

C_15_H_13_BrN_2_O_2_·CH_4_O
*M* *_r_* = 365.23
Monoclinic, 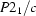

*a* = 13.585 (1) Å
*b* = 6.715 (1) Å
*c* = 18.377 (1) Å
β = 104.429 (2)°
*V* = 1623.5 (3) Å^3^
*Z* = 4
Mo *K*α radiation
μ = 2.55 mm^−1^
*T* = 298 K
0.20 × 0.20 × 0.17 mm

#### Data collection

Bruker SMART CCD area-detector diffractometer
Absorption correction: multi-scan (*SADABS*; Bruker, 2001 ▶) *T*_min_ = 0.630, *T*_max_ = 0.672
9539 measured reflections
3539 independent reflections
2132 reflections with *I* > 2σ(*I*)
*R*_int_ = 0.030

#### Refinement

*R*[*F* ^2^ > 2σ(*F* ^2^)] = 0.039
*wR*(*F* ^2^) = 0.103
*S* = 1.02
3539 reflections
205 parameters
1 restraint
H atoms treated by a mixture of independent and constrained refinement
Δρ_max_ = 0.41 e Å^−3^
Δρ_min_ = −0.52 e Å^−3^

### 

Data collection: *SMART* (Bruker, 2007 ▶); cell refinement: *SAINT* (Bruker, 2007 ▶); data reduction: *SAINT*; program(s) used to solve structure: *SHELXTL* (Sheldrick, 2008 ▶); program(s) used to refine structure: *SHELXTL*; molecular graphics: *SHELXTL*; software used to prepare material for publication: *SHELXTL*.

## Supplementary Material

Crystal structure: contains datablocks global, I. DOI: 10.1107/S1600536809030219/ci2872sup1.cif

Structure factors: contains datablocks I. DOI: 10.1107/S1600536809030219/ci2872Isup2.hkl

Additional supplementary materials:  crystallographic information; 3D view; checkCIF report

## Figures and Tables

**Table 1:** Hydrogen-bond geometry (Å, °)

*D*—H⋯*A*	*D*—H	H⋯*A*	*D*⋯*A*	*D*—H⋯*A*
O3—H3⋯O1	0.82	2.07	2.831 (3)	154
O3—H3⋯N2	0.82	2.60	3.211 (3)	132
N1—H1⋯O3^i^	0.90 (1)	2.12 (1)	2.993 (3)	166 (3)
C6—H6⋯O3^i^	0.93	2.49	3.406 (4)	168
C8—H8⋯O3^i^	0.93	2.56	3.370 (3)	146

Symmetry code: (i) 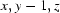.

**Fig. 1.**
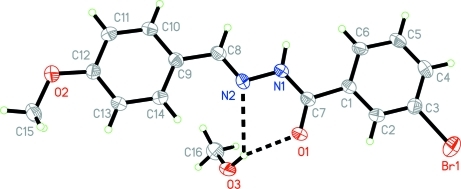
The asymmetric unit of the title compound. Displacement ellipsoids are drawn at the 30% probability level.

**Fig. 2.**
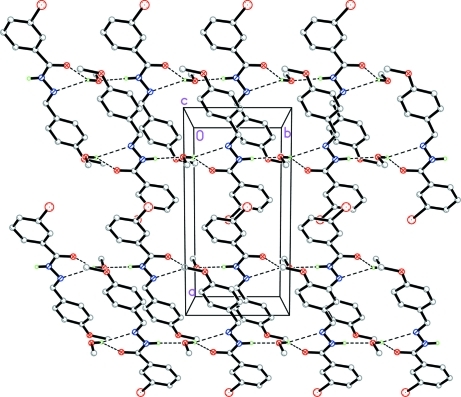
The crystal packing of the title compound, viewed along the c axis. Hydrogen bonds are shown as dashed lines. C-bound H atoms have been omitted for clarity.

## supplementary crystallographic information

### Comment

Study on the crystal structures of hydrazone derivatives is an interesting topic in structural chemistry. Recently, crystal structures of a number of hydrazone compounds have been reported (Mohd Lair *et al.*, 2009; Fun *et al.*, 2008; Li & Ban, 2009; Zhu *et al.*, 2009; Yang, 2007; You *et al.*, 2008). As a continuation of our work in this area (Qu *et al.*, 2008; Yang *et al.*, 2008; Cao & Lu, 2009a,b; Qu & Cao, 2009; Cao & Wang, 2009), the title new hydrazone compound derived from the reaction of 2-chlorobenzaldehyde with an equimolar quantity of 3-bromobenzohydrazide is reported.

The title compound (Fig. 1) consists of a hydrazone molecule and a methanol molecule of crystallization. The methanol molecule is linked to the hydrazone molecule through O—H···O and O—H···N hydrogen bonds (Table 1). The hydrazone molecule displays an *E* configuration about the C═N bond. The dihedral angle between the two benzene rings is 4.0 (2)°. In the crystal structure, molecules are linked through intermolecular N—H···O, O—H···O, O—H···N and C—H···O hydrogen bonds (Table 1) to form chains running along the *b* axis (Fig. 2).

### Experimental

The title compound was prepared by refluxing equimolar quantities of 4-methoxybenzaldehyde with 3-bromobenzohydrazide in methanol. Colourless block-like crystals were formed by slow evaporation of the solution in air.

### Refinement

Atom H1 was located in a difference Fourier map and refined isotropically, with the N-H distance restrained to 0.90 (1) Å. The other H atoms were placed in idealized positions and constrained to ride on their parent atoms, with a O-H distance of 0.82 Å, C-H distances of 0.93-0.96 Å, and with *U*_iso_(H) set at 1.2*U*_eq_(C) and 1.5*U*_eq_(methyl C).

### Crystal data

**Table:**

C_15_H_13_BrN_2_O_2_·CH_4_O	*F*(000) = 744
*M**_r_* = 365.23	*D*_x_ = 1.494 Mg m^−^^3^
Monoclinic, *P*2_1_/*c*	Mo *K*α radiation, λ = 0.71073 Å
Hall symbol: -P 2ybc	Cell parameters from 2168 reflections
*a* = 13.585 (1) Å	θ = 2.7–24.6°
*b* = 6.715 (1) Å	µ = 2.55 mm^−^^1^
*c* = 18.377 (1) Å	*T* = 298 K
β = 104.429 (2)°	Block, colourless
*V* = 1623.5 (3) Å^3^	0.20 × 0.20 × 0.17 mm
*Z* = 4	

### Data collection

**Table:**

Bruker SMART CCD area-detector diffractometer	3539 independent reflections
Radiation source: fine-focus sealed tube	2132 reflections with *I* > 2σ(*I*)
graphite	*R*_int_ = 0.030
ω scans	θ_max_ = 27.0°, θ_min_ = 1.6°
Absorption correction: multi-scan (*SADABS*; Bruker, 2001)	*h* = −17→17
*T*_min_ = 0.630, *T*_max_ = 0.672	*k* = −8→8
9539 measured reflections	*l* = −23→20

### Refinement

**Table:**

Refinement on *F*^2^	Primary atom site location: structure-invariant direct methods
Least-squares matrix: full	Secondary atom site location: difference Fourier map
*R*[*F*^2^ > 2σ(*F*^2^)] = 0.039	Hydrogen site location: inferred from neighbouring sites
*wR*(*F*^2^) = 0.103	H atoms treated by a mixture of independent and constrained refinement
*S* = 1.01	*w* = 1/[σ^2^(*F*_o_^2^) + (0.043*P*)^2^ + 0.516*P*] where *P* = (*F*_o_^2^ + 2*F*_c_^2^)/3
3539 reflections	(Δ/σ)_max_ = 0.001
205 parameters	Δρ_max_ = 0.41 e Å^−^^3^
1 restraint	Δρ_min_ = −0.52 e Å^−^^3^

### Special details

**Table:**

Geometry. All esds (except the esd in the dihedral angle between two l.s. planes) are estimated using the full covariance matrix. The cell esds are taken into account individually in the estimation of esds in distances, angles and torsion angles; correlations between esds in cell parameters are only used when they are defined by crystal symmetry. An approximate (isotropic) treatment of cell esds is used for estimating esds involving l.s. planes.
Refinement. Refinement of F^2^ against ALL reflections. The weighted R-factor wR and goodness of fit S are based on F^2^, conventional R-factors R are based on F, with F set to zero for negative F^2^. The threshold expression of F^2^ > 2sigma(F^2^) is used only for calculating R-factors(gt) etc. and is not relevant to the choice of reflections for refinement. R-factors based on F^2^ are statistically about twice as large as those based on F, and R- factors based on ALL data will be even larger.

### Fractional atomic coordinates and isotropic or equivalent isotropic displacement parameters (Å^2^)

**Table:**

	*x*	*y*	*z*	*U*_iso_*/*U*_eq_	
Br1	0.53424 (3)	0.09750 (6)	−0.18341 (2)	0.09185 (19)	
O1	0.26855 (15)	0.2642 (3)	−0.02385 (11)	0.0608 (5)	
O2	−0.19847 (15)	0.3318 (3)	0.21672 (11)	0.0595 (5)	
O3	0.20352 (18)	0.5573 (3)	0.06465 (12)	0.0676 (6)	
H3	0.2041	0.4585	0.0385	0.101*	
N1	0.21308 (16)	−0.0152 (3)	0.02088 (12)	0.0460 (5)	
N2	0.14486 (16)	0.0932 (3)	0.04946 (12)	0.0477 (5)	
C1	0.34289 (18)	−0.0397 (4)	−0.04807 (14)	0.0410 (6)	
C2	0.3955 (2)	0.0578 (4)	−0.09241 (14)	0.0470 (6)	
H2	0.3858	0.1937	−0.1011	0.056*	
C3	0.4622 (2)	−0.0442 (4)	−0.12395 (15)	0.0504 (7)	
C4	0.4778 (2)	−0.2432 (4)	−0.11248 (16)	0.0573 (8)	
H4	0.5230	−0.3111	−0.1341	0.069*	
C5	0.4257 (2)	−0.3414 (4)	−0.06842 (17)	0.0560 (7)	
H5	0.4360	−0.4773	−0.0602	0.067*	
C6	0.3584 (2)	−0.2429 (4)	−0.03613 (15)	0.0488 (7)	
H6	0.3236	−0.3120	−0.0065	0.059*	
C7	0.27195 (19)	0.0826 (4)	−0.01586 (14)	0.0446 (6)	
C8	0.0896 (2)	−0.0058 (4)	0.08234 (15)	0.0485 (6)	
H8	0.0978	−0.1432	0.0862	0.058*	
C9	0.01344 (18)	0.0883 (4)	0.11444 (14)	0.0430 (6)	
C10	−0.0341 (2)	−0.0209 (4)	0.15986 (15)	0.0488 (7)	
H10	−0.0179	−0.1549	0.1683	0.059*	
C11	−0.1040 (2)	0.0628 (4)	0.19264 (15)	0.0519 (7)	
H11	−0.1342	−0.0137	0.2232	0.062*	
C12	−0.12967 (19)	0.2614 (4)	0.18031 (14)	0.0451 (6)	
C13	−0.0856 (2)	0.3731 (4)	0.13385 (15)	0.0474 (6)	
H13	−0.1038	0.5058	0.1242	0.057*	
C14	−0.0142 (2)	0.2866 (4)	0.10182 (15)	0.0474 (6)	
H14	0.0159	0.3630	0.0711	0.057*	
C15	−0.2213 (2)	0.5384 (4)	0.21096 (18)	0.0662 (9)	
H15A	−0.1611	0.6134	0.2330	0.099*	
H15B	−0.2728	0.5674	0.2370	0.099*	
H15C	−0.2455	0.5742	0.1590	0.099*	
C16	0.2481 (3)	0.5111 (5)	0.13973 (19)	0.0749 (9)	
H16A	0.3160	0.4636	0.1445	0.112*	
H16B	0.2088	0.4098	0.1563	0.112*	
H16C	0.2500	0.6283	0.1700	0.112*	
H1	0.210 (2)	−0.1474 (16)	0.0258 (17)	0.080*	

### Atomic displacement parameters (Å^2^)

**Table:**

	*U*^11^	*U*^22^	*U*^33^	*U*^12^	*U*^13^	*U*^23^
Br1	0.1033 (3)	0.0942 (3)	0.0980 (3)	−0.0125 (2)	0.0627 (2)	0.0055 (2)
O1	0.0810 (14)	0.0302 (11)	0.0831 (14)	0.0074 (9)	0.0428 (11)	0.0037 (9)
O2	0.0673 (12)	0.0512 (11)	0.0699 (13)	0.0065 (9)	0.0358 (10)	0.0029 (10)
O3	0.1030 (16)	0.0346 (11)	0.0763 (15)	0.0028 (11)	0.0434 (13)	−0.0005 (10)
N1	0.0513 (13)	0.0327 (11)	0.0584 (14)	0.0025 (10)	0.0218 (11)	−0.0012 (11)
N2	0.0514 (13)	0.0417 (12)	0.0538 (14)	0.0046 (10)	0.0202 (11)	−0.0054 (11)
C1	0.0431 (14)	0.0351 (14)	0.0431 (15)	0.0001 (11)	0.0075 (12)	−0.0043 (11)
C2	0.0519 (16)	0.0369 (14)	0.0515 (16)	−0.0012 (12)	0.0112 (13)	0.0010 (12)
C3	0.0512 (16)	0.0540 (18)	0.0481 (16)	−0.0026 (13)	0.0163 (13)	−0.0029 (13)
C4	0.0560 (18)	0.0532 (18)	0.0635 (19)	0.0122 (14)	0.0167 (15)	−0.0099 (15)
C5	0.0638 (19)	0.0358 (15)	0.0686 (19)	0.0104 (13)	0.0172 (16)	−0.0023 (14)
C6	0.0544 (17)	0.0328 (14)	0.0599 (17)	0.0010 (12)	0.0153 (14)	0.0017 (12)
C7	0.0483 (15)	0.0376 (16)	0.0471 (15)	0.0051 (12)	0.0105 (12)	−0.0003 (12)
C8	0.0520 (17)	0.0381 (14)	0.0564 (17)	0.0032 (13)	0.0153 (14)	−0.0028 (13)
C9	0.0411 (14)	0.0406 (15)	0.0453 (15)	−0.0004 (12)	0.0071 (12)	−0.0041 (12)
C10	0.0567 (17)	0.0361 (14)	0.0548 (16)	0.0020 (12)	0.0161 (14)	0.0028 (13)
C11	0.0606 (18)	0.0443 (16)	0.0555 (17)	−0.0027 (13)	0.0231 (14)	0.0082 (13)
C12	0.0454 (15)	0.0463 (16)	0.0459 (15)	−0.0012 (12)	0.0158 (12)	−0.0027 (12)
C13	0.0538 (16)	0.0339 (14)	0.0559 (17)	0.0038 (12)	0.0165 (13)	0.0013 (12)
C14	0.0537 (16)	0.0397 (15)	0.0520 (16)	−0.0033 (12)	0.0189 (13)	0.0029 (13)
C15	0.073 (2)	0.059 (2)	0.073 (2)	0.0194 (16)	0.0303 (17)	−0.0001 (16)
C16	0.086 (2)	0.064 (2)	0.078 (3)	−0.0032 (18)	0.028 (2)	−0.0082 (19)

### Geometric parameters (Å, °)

**Table:**

Br1—C3	1.895 (3)	C6—H6	0.93
O1—C7	1.227 (3)	C8—C9	1.456 (3)
O2—C12	1.363 (3)	C8—H8	0.93
O2—C15	1.420 (3)	C9—C10	1.385 (4)
O3—C16	1.396 (4)	C9—C14	1.387 (3)
O3—H3	0.82	C10—C11	1.366 (4)
N1—C7	1.340 (3)	C10—H10	0.93
N1—N2	1.381 (3)	C11—C12	1.382 (4)
N1—H1	0.895 (10)	C11—H11	0.93
N2—C8	1.264 (3)	C12—C13	1.380 (3)
C1—C2	1.376 (3)	C13—C14	1.381 (3)
C1—C6	1.390 (4)	C13—H13	0.93
C1—C7	1.496 (3)	C14—H14	0.93
C2—C3	1.374 (3)	C15—H15A	0.96
C2—H2	0.93	C15—H15B	0.96
C3—C4	1.361 (4)	C15—H15C	0.96
C4—C5	1.370 (4)	C16—H16A	0.96
C4—H4	0.93	C16—H16B	0.96
C5—C6	1.376 (4)	C16—H16C	0.96
C5—H5	0.93		
			
C12—O2—C15	117.8 (2)	C10—C9—C14	117.5 (2)
C16—O3—H3	109.5	C10—C9—C8	119.9 (2)
C7—N1—N2	118.3 (2)	C14—C9—C8	122.6 (2)
C7—N1—H1	126 (2)	C11—C10—C9	121.8 (2)
N2—N1—H1	115 (2)	C11—C10—H10	119.1
C8—N2—N1	116.1 (2)	C9—C10—H10	119.1
C2—C1—C6	118.7 (2)	C10—C11—C12	120.0 (2)
C2—C1—C7	117.0 (2)	C10—C11—H11	120.0
C6—C1—C7	124.4 (2)	C12—C11—H11	120.0
C3—C2—C1	120.4 (2)	O2—C12—C13	124.9 (2)
C3—C2—H2	119.8	O2—C12—C11	115.5 (2)
C1—C2—H2	119.8	C13—C12—C11	119.6 (2)
C4—C3—C2	121.2 (2)	C12—C13—C14	119.6 (2)
C4—C3—Br1	119.9 (2)	C12—C13—H13	120.2
C2—C3—Br1	118.9 (2)	C14—C13—H13	120.2
C3—C4—C5	118.7 (2)	C13—C14—C9	121.5 (2)
C3—C4—H4	120.7	C13—C14—H14	119.3
C5—C4—H4	120.7	C9—C14—H14	119.3
C4—C5—C6	121.3 (3)	O2—C15—H15A	109.5
C4—C5—H5	119.3	O2—C15—H15B	109.5
C6—C5—H5	119.3	H15A—C15—H15B	109.5
C5—C6—C1	119.6 (3)	O2—C15—H15C	109.5
C5—C6—H6	120.2	H15A—C15—H15C	109.5
C1—C6—H6	120.2	H15B—C15—H15C	109.5
O1—C7—N1	122.5 (2)	O3—C16—H16A	109.5
O1—C7—C1	120.5 (2)	O3—C16—H16B	109.5
N1—C7—C1	117.0 (2)	H16A—C16—H16B	109.5
N2—C8—C9	122.1 (2)	O3—C16—H16C	109.5
N2—C8—H8	118.9	H16A—C16—H16C	109.5
C9—C8—H8	118.9	H16B—C16—H16C	109.5

### Hydrogen-bond geometry (Å, °)

**Table:**

*D*—H···*A*	*D*—H	H···*A*	*D*···*A*	*D*—H···*A*
O3—H3···O1	0.82	2.07	2.831 (3)	154
O3—H3···N2	0.82	2.60	3.211 (3)	132
N1—H1···O3^i^	0.90 (1)	2.12 (1)	2.993 (3)	166 (3)
C6—H6···O3^i^	0.93	2.49	3.406 (4)	168
C8—H8···O3^i^	0.93	2.56	3.370 (3)	146

Symmetry codes: (i) *x*, *y*−1, *z*.
